# Novel Mat Exergaming to Improve the Physical Performance, Cognitive Function, and Dual-Task Walking and Decrease the Fall Risk of Community-Dwelling Older Adults

**DOI:** 10.3389/fpsyg.2020.01620

**Published:** 2020-07-24

**Authors:** Hsien-Te Peng, Cheng-Wen Tien, Pay-Shin Lin, Hsuen-Ying Peng, Chen-Yi Song

**Affiliations:** ^1^Department of Physical Education, Chinese Culture University, Taipei, Taiwan; ^2^Department of Exercise and Health Science, National Taipei University of Nursing and Health Sciences, Taipei, Taiwan; ^3^Department of Physical Therapy and Graduate Institute of Rehabilitation Science, College of Medicine, Chang Gung University, Taoyuan, Taiwan; ^4^Master Degree Program in Healthcare Industry, Chang Gung University, Taoyuan, Taiwan; ^5^Health Aging Research Center, Chang Gung University and Chang Gung Memorial Hospital, Taoyuan, Taiwan; ^6^Department of Long-Term Care, National Taipei University of Nursing and Health Sciences, Taipei, Taiwan

**Keywords:** elderly, exergame, fall prevention, smart exercise, combined physical, cognitive training

## Abstract

Physical exercise and cognitive training were previously demonstrated to improve the physical functioning and decrease the incidence of falls for older adults. This study aimed to utilize an interactive exergame mat system to develop a novel cognitive–physical training program and explore the training effects on physical performance, cognitive function, dual-task walking (DTW), and fall risk compared to the control condition. In this quasi-experimental non-randomized controlled intervention study, 110 community-dwelling older adults participated. The exercise group (*n* = 56; mean age, 70.7 ± 4.6 years) performed ladder-type, three-by-three grid-type, and circle-type mat exergames with simultaneous cognitive–physical training (EMAT), while the control group (*n* = 54; mean age, 72.0 ± 5.7 years) underwent a multicomponent exercise intervention focused on physical and cognitive training. A 2 h training session was completed weekly for 3 months. Functional fitness (including upper- and lower-extremity strength and flexibility, grasp strength, aerobic endurance, static balance, dynamic balance and agility), a foot tapping test (FTT), the Montreal Cognitive Assessment (MoCA), DTW, and a fall risk questionnaire (FRQ) were assessed before and after the interventions. The EMAT program enhanced upper-extremity strength, lower-extremity strength and flexibility, aerobic endurance, and dynamic balance and agility; improved DTW and FTT performances; and decreased FRQ score. EMAT also showed a significant advantage over control in terms of lower-extremity strength and flexibility, aerobic endurance, dynamic balance and agility, and FRQ score (all *P* < 0.05). The current study provides evidence of the effects of a novel mat exergaming program on physical and cognitive performance. EMAT effectively reduced the fall risk and increased the dual-task ability of walking, factors that are important in fall prevention for community-dwelling older adults.

## Introduction

Population aging is increasing the demand for health and social care services, and falls create an enormous burden (Stevens et al., 2006). Research indicated that about one-third of community-dwelling older people experience at least one fall per year (Rubenstein, 2006). Falls are associated with greater morbidity and mortality rates, reduced overall functioning, and early admission to long-term care facilities (Tinetti, 1986; Rubenstein et al., 1994; Brown, 1999). Therefore, fall prevention is an important global health issue in our aged population.

Fall interventions through exergame technologies are emerging. A systemic review of 25 studies of exergame technology and interactive interventions for fall prevention in older populations revealed that exergame interventions improved physical and cognitive functioning in elderly individuals (Choi et al., 2017). A meta-analysis of 18 randomized controlled trials of active video games for improving physical performance in community-dwelling older people indicated that active video games can improve mobility and balance when used alone or as part of an exercise program (Taylor et al., 2018). Dance exergaming, a motor–cognitive dual-task training method that challenged balance with stimulus characteristics of game play (Smith et al., 2011), was shown to improve dual-task walking (DTW) (de Bruin et al., 2011; Pichierri et al., 2012; Eggenberger et al., 2015). A recent study further investigated the effects of dance exergaming in fallers and non-fallers among community-dwelling older women and demonstrated the benefits of training on physical and psychological aspects. The authors concluded that dance exergaming can be indicated to decrease depressive symptoms in fallers and increase the peak torque (of the hamstrings) in non-fallers among community-dwelling older women (Rodrigues et al., 2018). The aforementioned evidence led to support of the use of exergame-based interventions for community-dwelling older people.

According to the American College of Sports Medicine and the American Heart Association (Nelson et al., 2007; American College of Sports Medicine et al., 2009), recommended physical activity and exercise for adults 65 years and older include multicomponent exercises involving strength, aerobic/endurance, flexibility, and balance/coordination training. To reduce the risk of fall-related injury, community-dwelling older adults with substantial risk of falls should perform exercises that maintain or improve balance. However, commercial systems developed specifically to increase balance in the elderly are rare. The training focus of some exergames such as Wii Sports and dance exergaming, for example, was mainly the upper and lower limbs, respectively. Adcock et al. (2019) developed a multicomponent exergame for older adults including Tai Chi–inspired exercises, dance movements, and step-based cognitive games to train strength, balance, and cognition, but their exergame system was developed with the purpose of training in the home-based setting. Furthermore, most exergames were not performed as group exercises. Providing opportunities for older adults to interact with others via playing collaborative or competitive exergames can enhance their social well-being such as reduction of loneliness, increased social connection, and positive attitudes toward others (Li et al., 2018).

The purposes of this study were to utilize an interactive exergame mat system to develop a novel cognitive–physical training program with multicomponent exercises and explore the training effects on physical performance, cognitive function, DTW, and fall risk compared to the control condition.

## Materials and Methods

### Study Design and Participants

This quasi-experimental non-randomized controlled intervention study included two groups of community-dwelling older adults who completed 3-months exercise training interventions carried out by experienced physical therapists. Assessments were performed before and after the interventions by trained assessors who were blinded to the group assignment. The study protocol was approved by the ethics committee of the local hospital. Participants were recruited from local community activity centers in Taipei and New Taipei City through advertisements. The inclusion criteria were: (1) being aged 65 years or older and (2) being able to walk independently without any assistive devices. The exclusion criteria included: (1) severe lower-extremity joint pain and (2) the presence of cognitive impairment, or visual problems that impeded their participation in the study. All participants provided signed informed consent prior to participating. The trial was registered at ClinicalTrials.gov (NCT04284709).

### Interventions

**FIGURE 1:**
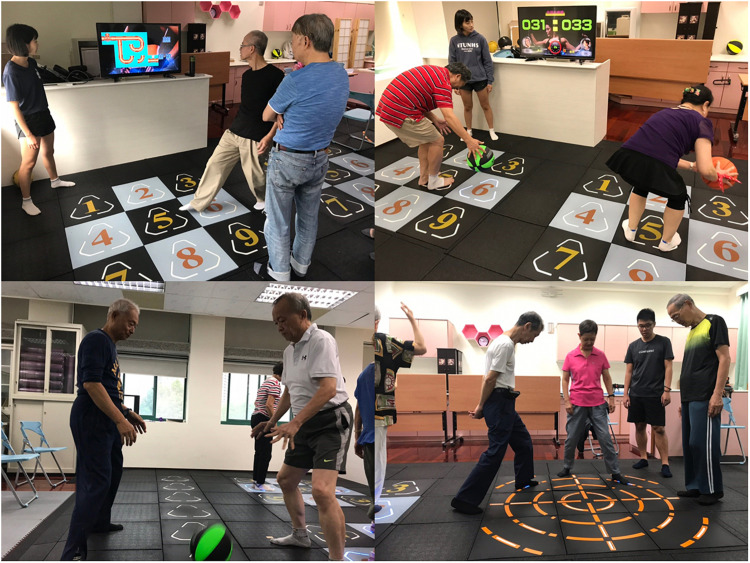
Example of mat exergame with simultaneous cognitive–physical training. **(A)** Participants performing steps on the three-by-three grid-type mat to play a puzzle game. **(B)** Participants competing against each other to hit each light on the grid to turn it “off” the most quickly. **(C)** Participants walking sideways to play a reaction time game with a basketball (turning the light “off” in a random order) on the ladder-type mat. **(D)** Participants performing verbal memory exercises on the circle-type mat for balance and agility training.

A 2 h training session, including 20 min of warm-up, 90 min of main activity, and 10 min of cool-down, was completed weekly for a total of 3 months. The exercise group completed a ladder-type, three-by-three grid-type, and circle-type mat exergame (Stampede, Compal Electronics Inc., Taipei, Taiwan) with simultaneous cognitive–physical training (EMAT; Figure 1), while the control group completed a conventional multicomponent exercise intervention focused on physical and cognitive training (Table 1). A TheraBand and Swiss ball were applied to assist the resistance training of both the upper and lower extremities and trunk muscles. Dance-based exercise was performed for aerobic training. Balance exercises included static (e.g., standing) and dynamic (e.g., walking) balance training and task-oriented exercise. Training that targeted cognitive abilities was integrated into the physical training program or the group activities.

**TABLE 1:** Multicomponent exercise intervention programs focusing on physical and cognitive training.

**Week**	**Exercise group (EMAT exercise)**	**Control group (Conventional exercise)**
1	Course introduction and pre-test	Course introduction and pre-test
2	Ladder: straight walk, side walk, and reaction time game with mat sparkled in a random sequence (entry level)	Muscle strengthening (TheraBand)
	Grid: grid-step aerobics	Shape recognition
	Circle: static balance training, multi-directional excursion balance and coordination training (entry level)	
3	Ladder: straight walk, side walk, and reaction time game (advanced level, with various foot steps)	Muscle strengthening (Swiss ball)
	Grid: numerical-step aerobics	Pattern recognition
	Circle: static balance training, multi-directional excursion balance and coordination training (advanced level)	
4	Ladder: v-step aerobics (entry level)	Muscle strengthening (TheraBand)
	Grid: seated abdominal crunch with ball between knees or ankles (entry level)	Reaction training
	Circle: multi-directional lunge exercise, star excursion balance training (entry level)	
5	Ladder: v-step aerobics (advanced level, using complicated movement with cognitive manipulation)	Muscle strengthening (Swiss ball)
	Grid: seated abdominal crunch (advanced level)	Fine motor training
	Circle: multi-directional lunge exercise with TheraBand, star excursion balance training (advanced level)	
6	Ladder: mini-squat walking, reaction time game using basketball (single play)	Aerobic exercise (entry level)
	Grid: stepping reaction game, and with concurrent calculation of addition (collaborative play)	Numerical cognition
	Circle: boxing aerobics with cross-shaped steps (entry level)	
7	Ladder: mini-squat walking with a TheraBand on the thighs, reaction time game using basketball (collaborative play)	Aerobic exercise (entry level) Character and color recognition
	Grid: stepping reaction game with concurrent calculation of subtraction (collaborative play)	
	Circle: boxing aerobics with cross-shaped steps (advanced level)	
8	Ladder: aerobics with high knee lift, plank exercise for core strengthening (entry level)	Aerobic exercise (advanced level)
	Grid: puzzle game and reaction time game using basketball to hit #1–#9 for three rounds (single or collaborative play)	Memory training
	Circle: agility training (entry level)	
9	Ladder: aerobics with high knee lift, plank exercise for core strengthening (advanced level)	Aerobic exercise (advanced level)
	Grid: puzzle game and reaction time game using basketball to hit #4 and #6 alternatively while single-leg stand on #5	Memory training
	Circle: agility training (advanced level)	
10	Ladder: comprehensive exercise combined training (multiple players)	Balance exercise (entry level)
	Grid: back-to-back seated core strengthening game using half twist to pass the basketball to hit the mat (collaborative play)	Fine motor training
	Circle: aerobics with diagonal (X) steps	
11	Ladder: comprehensive exercise combined training (multiple players)	Balance exercise (advanced level)
	Grid: team competition games	Cognitive strategies
	Circle: aerobics with diagonal (X) steps	
12	Feedback and post-test	Feedback and post-test

*Multicomponent exercises included strength, aerobic endurance, flexibility, and balance training. The 2 h training session included 20 min of warm-up, 90 min of main activity, and 10 min of cool-down. Active range of motion and stretching exercises were integrated in the warm-up and cool-down phases. The 90 min of physical and cognitive training was divided into two to three sessions with 5–10 min of rest in between. EMAT = mat exergames with simultaneous cognitive–physical training.*

The physical training programs were created based on current recommendations for physical fitness and fall prevention for elderly persons, including strength, aerobic endurance, flexibility, and balance exercises (Nelson et al., 2007; American College of Sports Medicine et al., 2009). The exercise group received multicomponent physical training on a weekly basis, while the control group received one of strength, aerobic endurance, or balance exercise per week. Training that targeted cognitive abilities was delivered via exergame technologies to the exercise group and cognitive game playing to the control group.

### Outcome Measures

The main outcome measurements included physical performance, cognitive function, DTW, and fall risk.

#### Functional Fitness

The Senior Fitness Test was used for assessing various dimensions of functional fitness, including a 30 s chair stand test for lower-limb muscle strength, 30 s arm curl test for upper-limb muscle strength, 2 min step test for aerobic endurance, chair sit and reach test for lower-body flexibility, back scratch test for upper-body flexibility, and 2.44 m up and go test for agility and dynamic balance. The test–retest reliability of the Senior Fitness Test was reported to be high to very high in a normal older population [intraclass correlation coefficient (ICC) = 0.81–0.96] (Rikli and Jones, 2013). All tests were evaluated following the testing procedure in the test manual (Rikli and Jones, 2013). In addition, the single-leg stand test and grasp strength were measured because one-leg balance is an important predictor of injurious falls in older persons (Vellas et al., 1997), while handgrip strength is used to screen for sarcopenia (Chen et al., 2014) and found to be significantly associated with falls (Yang et al., 2018). The single-leg stand test was administered with participants standing on their dominant leg and holding the one-legged stance while keeping their hands on the waist and the foot lifted off the floor. The test was terminated following a maximum of 30 s. The handgrip strength of participants’ dominant hands was assessed using a hand dynamometer (TTM-YD, Tokyo, Japan). Participants were asked to stand with their arms by the side of the body and were given verbal encouragement to give their maximum effort. The test–retest reliability of the single-leg stand test (ICC = 0.86) and grasp strength test (ICC = 0.94–0.98) was good to excellent in community-dwelling older adults (Schaubert and Bohannon, 2005; Goldberg et al., 2011). All tests but the 30 s chair stand, 30 s arm curl, and 2 min step tests were measured twice, and the best value of each test was included in further analysis.

#### Foot Tapping Test

The foot tapping test (FTT), with good reliability (ICC = 0.79) (Hinman, 2019), is a simple test used to examine lower-limb motor function. The participant was seated on a chair with the hips and knees at ∼90 degrees and asked to tap the sole of the foot on the floor as many times as possible within a 10 s duration while keeping the heel in contact with the ground (Numasawa et al., 2012). Two feet were tested randomly twice, and the best value of each foot was included in further analysis.

#### Montreal Cognitive Assessment

The Montreal Cognitive Assessment (MoCA), with good reliability (Cronbach’s α = 0.86 and ICC = 0.88) and validity (*r* = 0.91 between MoCA scores and Mini-Mental State Examination scores) (Tsai et al., 2012), was used to assess cognitive function including attention and concentration, executive function, memory, language, visuoconstructional skills, conceptual thinking, calculations, and orientation. Task performance was summed to generate a total score; a score of 26 or less was indicative of cognitive impairment. An educational correction of 1 point is added to the total score for individuals with 12 or fewer years of education (Tsai et al., 2012).

#### Dual-Task Walking

In line with a previous study (Liao et al., 2019), the DTW testing protocol comprised two conditions: (1) walking while counting backward in increments of three from a random number between 90 and 100 (cognitive DTW, DTW_C) and (2) walking while carrying a tray (size: 38 cm × 28 cm × 5 cm) that was 80% full of water (motor DTW, DTW_M). The primary walking task was the 2.44 m up and go, and participants were asked to get up from a seated position, walk 2.44 m at their fast speed, turn, and return to seated position. Each test condition was conducted twice; if a participant stopped walking or performing the cognitive task, the trial was repeated, and the best value was included in further analysis. The test–retest reliability of the DTW_C and DTW_M was good (ICC = 0.85–0.88).

#### Fall Risk Questionnaire

The self-reported fall risk questionnaire (FRQ), which was developed based on evidence and clinical acceptability and incorporates the strongest evidence-based fall risk factors, has good concurrent validity (Rubenstein et al., 2011). It offers a feasible way to identify at-risk individuals in community settings. The FRQ contains 12 items that evaluate risk factors associated with falls, i.e., history of previous falls, fear of falling, gait/balance disturbances, muscle weakness, incontinence, sensation and proprioception, depression, vision, and medications. A total score of ≥4 points indicated a fall risk on the FRQ (Rubenstein et al., 2011).

#### Data Analyses

Statistical analyses were performed using SPSS version 23.0 (IBM, Armonk, NY, United States). The data were subjected to an intention-to-treat analysis and included all dropouts with the last observation carried forward. The sample size was calculated using G^∗^Power 3.1 software. At least 51 participants in each group were required based on assumptions of an effect size of 0.5, an alpha of 0.05, and a statistical power of 80%.

Descriptive statistics were used to determine the participants’ demographics and their physical performance, MoCA score, DTW performance, and FRQ score. Data normality was examined by Kolmogorov–Smirnov test. The group differences in the baseline measurements were analyzed using independent *t*-tests. Two-way analysis of variance (ANOVA) with repeated measures was used to determine the effects of the intervention. Due to the group differences in the baseline measurements, one-way analysis of covariance (ANCOVA) was applied with the following dependent variables: MoCA score, chair stand, back scratch, 2 min step, and 2.44 m up and go. The level of significance was set at α = 0.05.

## Results

### Descriptive Statistics

A total of 110 community-dwelling older adults were recruited. Among them, 56 participated in the exercise group, and 54 participated in the control group. The demographic data for the study participants are presented in Table 2. The men in the exercise group and control group were similar in age (71.2 ± 3.8 vs 74.0 ± 7.9, *p* = 0.44) and body mass index (23.62 ± 2.38 vs 21.97 ± 3.59, *p* = 0.22). The women in the exercise group and control group were also similar in age (70.5 ± 4.9 vs 71.8 ± 5.4, *p* = 0.26) and body mass index (23.71 ± 3.38 vs 24.84 ± 3.76, *p* = 0.15).

**TABLE 2:** Demographic characteristics of the study participants.

**Variable**	**Exercise group EMAT exercise (n = 56)**	**Control group Conventional exercise (*n* = 54)**
Age (years)	70.74.6	72.05.7
Gender (women/men)	39/17	48/6
Height (cm)	158.36.9	155.06.2
Weight (kg)	59.48.7	58.89.2
Body mass index (BMI, kg/m^2^)	23.683.10	24.523.82

### Training Effects

**FIGURE 2:**
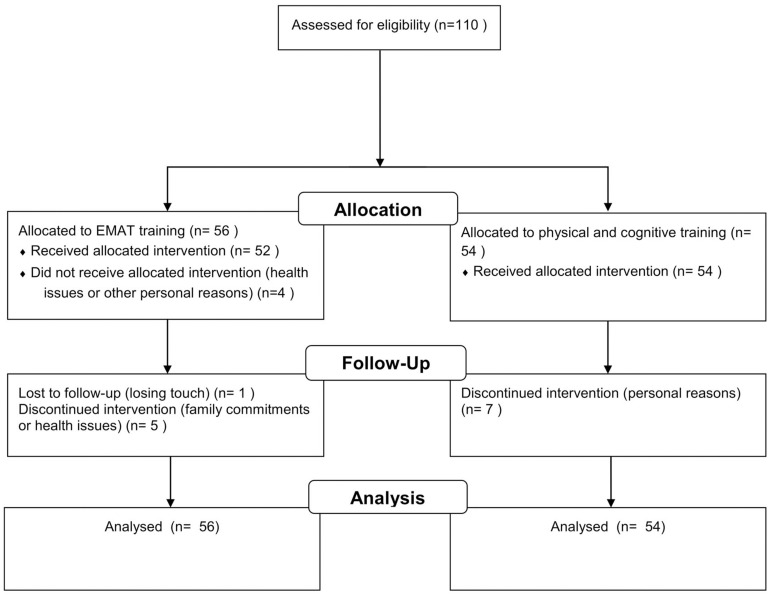
Flowchart of the present study.

A total of 93 participants (46 in the exercise group and 47 in the control group) completed the training. The final analysis included all dropouts (Figure 2). The variables of interest at pre-intervention are similar between two groups except for the MoCA score, chair stand, back scratch, 2 min step, and 2.44 m up and go (all *p* < 0.05).

The treatment effects are summarized in Table 3. There were significant group-by-time interaction effects on fall risk (FRQ) (*F* = 5.72, *p* = 0.018) and chair sit and reach (*F* = 11.17, *p* = 0.001). The EMAT group showed greater improvement compared to the control group. Statistically significant time effects were found in arm curl (*F* = 17.17, *p* < 0.001), FTT (Rt: *F* = 10.74, *p* = 0.001; Lt: *F* = 6.85, *p* = 0.01), and DTW_M (*F* = 10.04, *p* = 0.002) performances. A time effect was noted in DTW_C, with borderline significance (*F* = 3.67, *p* = 0.058). One-way ANCOVAs showed statistically significant differences in post-intervention chair stand (*F* = 12.89, *p* = 0.001), 2 min step (*F* = 40.31, *p* < 0.001), and 2.44 m up and go (*F* = 18.92, *p* < 0.001) between the two groups when adjusted for pre-intervention values. The performances were significantly better in the EMAT group than in the control group (Table 3).

**TABLE 3:** Pre- and post-intervention changes in physical performance, cognitive function, dual-task walking, and fall risk in the exercise versus control group.

	**Exercise group (EMAT exercise) (*n* = 56)**		**Control group (conventional exercise) (*n* = 54)**	
	**Pre-intervention**	**Post-intervention**	**Pre-intervention**	**Post-intervention**
Fall risk questionnaire (FRQ)^a^	1.842.16	1.432.13	2.332.35	3.114.08
Montreal Cognitive Assessment (MoCA)	27.042.88	27.552.67	21.113.46	23.853.76
Chair stand (no. of stands)^c^	21.756.85	24.046.43	18.854.15	18.985.15
Arm curl (no. of reps)^b^	19.164.97	21.504.90	19.334.56	20.395.58
Back scratch (cm)	0.3010.87	1.179.89	−4.8212.16	−3.3310.82
Chair sit and reach (cm)^a^	10.1810.82	13.7110.03	7.0610.22	5.969.16
2-min step (no. of steps)^c^	116.9616.69	122.5714.97	97.7237.86	94.4825.73
2.44 m up and go (s)^c^	5.961.21	5.210.92	6.741.73	6.912.24
Motor dual-task walking (DTW_M, s)^b^	8.021.69	7.191.51	8.482.30	8.003.11
Cognitive dual-task walking (DTW_C, s)	8.192.49	7.112.39	9.696.26	9.094.65
Single-leg stand (s)	23.089.57	25.507.81	19.6110.28	19.9610.51
Handgrip strength (kg)	26.426.94	26.227.30	24.885.19	23.885.32
Rt foot tapping test (no. of reps)^b^	31.759.00	36.1611.04	32.706.22	34.307.88
Lt foot tapping test (no. of reps)^b^	31.208.26	35.0410.98	32.566.30	33.177.44

*^*a*^Significant group-by-time interaction effect. ^*b*^Significant time effect. ^*c*^Significant post-intervention differences after adjustment for pre-intervention values.*

## Discussion

The current study aimed to investigate the effects of EMAT training, a novel cognitive–physical training program, on the physical performance, cognitive function, DTW, and fall risk of community-dwelling older adults. The results indicated that this novel EMAT training showed benefits for community-dwelling older adults in terms of physical performance (including functional fitness and FTT performance), DTW, and fall risk. In particular, superiority was seen in lower-body strength and flexibility, aerobic endurance, dynamic balance and agility, and fall risk (FRQ). Although the EMAT training increased the MoCA score, the improvement in MoCA score compared to that in the control group was not different, perhaps due to the ceiling effect, since the EMAT group had high baseline cognitive function. It should be noted that the positive effects of EMAT training may be underestimated, since our analysis included all dropouts, with the last observation carried forward.

Physical exercise and cognitive training were previously demonstrated to improve physical functioning, walking capacity, and DTW as well as reduce the risk of falls for older adults (de Bruin et al., 2011; Pichierri et al., 2012; Theill et al., 2013; van het Reve and de Bruin, 2014; Eggenberger et al., 2015; Corregidor-Sánchez et al., 2020). The overall effects of the EMAT training for improving functional fitness, improving DTW, and reducing the fall risk were consistent with those of previous studies. Video game dancing, as a commonly used simultaneous cognitive–physical training, resulted in an increased walking speed in the fast DTW condition compared to strength and balance training alone (Pichierri et al., 2012) and reduced the dual-task costs of walking compared to usual care physical training (de Bruin et al., 2011). Eggenberger et al. (2015) further demonstrated that virtual reality video game dancing and treadmill walking with verbal memory exercise had a significant advantage versus a treadmill walking exercise in the dual-task factors of step time variability during fast walking. The authors also found that dance and memory tasks showed training-specific gait adaptations or transfers that reduced step time during fast walking and reduced gait variability during the dual-task exercise at the preferred walking speed, respectively (Eggenberger et al., 2015). Furthermore, exergame and balance training were shown to modulate prefrontal brain activity during walking and enhance executive function in older adults (Eggenberger et al., 2016). Hence, in our simultaneous cognitive–physical training, the EMAT program, the dance, memory, and balance task components resulted in an increased fast DTW walking speed, and a rapid step (FTT) was imaginable. Thus, significant improvements in DTW may be attributed to improvements in cognitive function and walking capacity.

Dance has been shown to be an effective training for fall prevention in the elderly (Kattenstroth et al., 2013). A systematic review and meta-analysis showed that both reactive and volitional stepping interventions reduced falls among older adults by improving reaction time, gait, balance, and balance recovery (Okubo et al., 2017). Beyond dance and memory tasks, the EMAT program utilized three types of mat exergames and incorporated diverse muscular strength, aerobics, and balance training movements with mat interaction. Participants practiced multi-directional changes (e.g., anteroposterior, mediolateral, and rotational moves) in body movements, squats, spins, and continuous steps and coordinated their attention and movement to react to the mat. The EMAT program not only involved lower-extremity training; it included upper-extremity training with powerful boxing movements, resistive rubber bands, and ball hitting. Trunk stabilization was achieved through exergaming with the mat. Furthermore, our novel mat exergame integrated multi-aspect cognitive training such as calculation, logical thinking and problem solving (e.g., puzzle games), visuomotor integration (e.g., basketball game), social interaction (e.g., multiple-player training), and a combination thereof. Taking these together, this innovative approach was feasible for community-dwelling older adults, and the training benefits and superiority were obvious compared to conventional multicomponent exercise interventions focused on physical and cognitive training.

Functional fitness plays an important role in fall prevention for the elderly. The 2.44 m up and go test, for instance, is commonly used to discriminate between community-residing older adult fallers and non-fallers (Rose et al., 2002). Zhao and Chung (2016) found that older adults at risk of falling have lower overall functional fitness, especially in terms of agility and dynamic balance, and aerobic endurance compared to those without a risk of falling. In this study, we measured functional fitness and FRQ and subsequently found that EMAT training resulted in significant improvements of both. These findings support previous evidence that multicomponent exercises effectively reduced the risk and incidence of falls (Gardner et al., 2000; Chang et al., 2004; Sherrington et al., 2011; Gillespie et al., 2012).

### Limitations

This study had some limitations. First, this was not a randomized controlled trial, and few elderly men participated in the conventional exercise group. Such a sex-based disparity, however, is usually noticed in community-based exercise programs in Taiwan. Based on the current study results, the EMAT, an effective exergame technology and interactive intervention, could offer an alternative to traditional exercise programs for maintaining or enhancing multiple functional abilities and could probably motivate elderly men to exercise for enjoyment. Second, multicomponent simultaneous cognitive–physical training was implemented, but the exact benefits from each EMAT training component were unpredictable. Third, we did not conduct follow-up measurements; hence, the feasibility of long-term maintenance of EMAT training gains remains uncertain. Finally, the exercise training frequency was only once weekly. Future studies are needed to accumulate evidence-based information that can inform best practices.

## Conclusion

The current study provided evidence of the effect of a novel mat exergaming program on physical and cognitive performance. EMAT effectively reduced the fall risk and increased the DTW ability, which is important in fall prevention for older adults.

## Data Availability Statement

The datasets generated for this study are available on request to the corresponding author.

## Ethics Statement

The studies involving human participants were reviewed and approved by Antai Medical Care Cooperation Antai-Tian-Sheng Memorial Hospital Institutional Review Board. The patients/participants provided their written informed consent to participate in this study. Written informed consent was obtained from the individual(s) for the publication of any potentially identifiable images or data included in this article.

## Author Contributions

C-YS conceived, designed the experiments, and drafted the manuscript. C-YS, H-TP, C-WT, and H-YP conducted the data acquisition. C-YS, H-TP, C-WT, and P-SL performed statistical analyses and interpretation of the data. All authors gave final approval for the version to be submitted.

## Conflict of Interest

The authors declare that the research was conducted in the absence of any commercial or financial relationships that could be construed as a potential conflict of interest.
